# Reliability and validity of intraoral and extraoral scanners

**DOI:** 10.1186/s40510-015-0108-7

**Published:** 2015-10-27

**Authors:** Helder B. Jacob, Graydon D. Wyatt, Peter H. Buschang

**Affiliations:** Department of Orthodontics, Texas A&M University Baylor College of Dentistry, 3302 Gaston Avenue, 75246 Dallas, TX USA

## Abstract

**Background:**

This study evaluated the reliability and validity of one extraoral [Ortho Insight 3D™ (Motionview Software, Hixson, TN/USA)] and two intraoral [ITero™ (Align Technologies, San Jose, CA/USA) and Lythos™ (Ormco Corp., Orange, CA/USA)] scanners.

**Methods:**

Fifteen dry human mandibles were scanned twice with each of the scanners, and digital models were generated. Five measurements were made on the dry mandibles and on each of the generated models, including intermolar width, intercanine width, posterior arch length, premolar crown diameter, and canine height. Systematic and random errors were evaluated based on replicate analyses. Differences were assessed using paired Student’s *t* tests.

**Results:**

Replicate analyses showed statistically significant systematic errors for only one measure (intermolar width measured from Ortho Insight 3D scans). Measurements taken from all three scanners were highly reliable, with intraclass correlations ranging from .926 to .999. Method errors were all less than 0.25 mm (averaged ≈0.12 mm). Posterior arch length and canine height were significantly smaller when measured on the Ortho Insight 3D scans than when measured on the dry mandibles and significantly smaller than when measured from the ITero and Lythos models.

**Conclusions:**

While all three scanners produced reliable measures, Ortho Insight 3D systematically underestimated arch length and canine height.

## Background

Plaster models that have been traditionally used in orthodontics for evaluating patients’ occlusal status have several limitations. They are subject to physical and chemical damage and they wear when repeatedly measured. Models can also distort over time due to variation of humidity and temperature [1, 2]. Plaster models are also costly, both in terms of the time required for the impressions, model fabrication, and model storage. To solve these problems, digital models were introduced in the late 1990s.

OrthoCAD™ was the first company to introduce digital models. They allowed orthodontists to store casts electronically, eliminate impressions, and minimize many of the limitations associated with plaster models [3]. Since the introduction of the first digital models to the orthodontic community in 1999, the technology has improved and numerous in-office dental scanners have been introduced. By 2014, digital models were being used for diagnostic purposes in 55 % of Pacific orthodontic practices and 21 % of Northeast practices [4].

Digital models are produced by digitizing the oral structures, either directly or indirectly, with intra- or extraoral scanners, respectively. Three types of scanners are typically used: mechanical scanners with a touch-probe, laser scanners, or white light scanners [5, 6]. Because light scanners work without touching the areas of interest, they are preferred over the touch-probe scanners [5]. There are four types of imaging technologies employed: triangulation, parallel confocal, accordion fringe interferometry, and 3D in motion video [7]. To gather surface data points, the light (laser or white) is projected from the source onto an object and either reflected back to a sensor or to an absorbing source. While a high-resolution image is an important requisite for accurately measuring dimensions, the mathematical models and algorithms used to reconstruct the model and produce the 3D images also play an important role [8, 9].

Before a scanner can be accepted by the orthodontic community, it has to be shown that it provides a valid and reliable representation of the dentoalveolar structures. Digital models produced with extraoral scanners have been shown to be valid when compared to direct measurement on plaster models, with the differences between the approaches considered to be clinically acceptable [10–17]. Intraoral scanners have also been shown to produce valid and reliable digital models [18, 19], but differences exist. For example, Flugge and coworkers [20] found that scanning with the ITero is less accurate than scanning with the D250.

The purpose of this study was to compare the reliability and validity of one extraoral scanner (Ortho Insight 3D™) and two intraoral (ITero™ and Lythos™) scanners. The validity and reliability of the Lythos has not been established, and intra- and extraoral scanners have not previously been compared.

## Methods

### Sample and measurements

The sample was comprised of 15 dried human adult mandibles from the Texas A&M University Baylor College of Dentistry Department of Biomedical Science. The mandibles had to be in good condition, with all of the teeth (from second molar to second molar) present. For each mandible, three sets of digital models were produced using three different scanning protocols, including one extraoral [Ortho Insight 3D (Motionview Software, Hixson, TN)] and two intraoral [ITero (Align Technologies, San Jose, CA) and Lythos (Ormco Corp., Orange, CA)]. For the Ortho Insight 3D scans, the mandibles were placed in the scanner and secured with double-sided tape. The two intraoral scans were performed using the manufacturers’ suggested protocols. Each mandible was scanned twice, as least 1 week apart, with each of the three scanners and Standard Tessellation Language (STL) files were created.

**Fig. 1:**
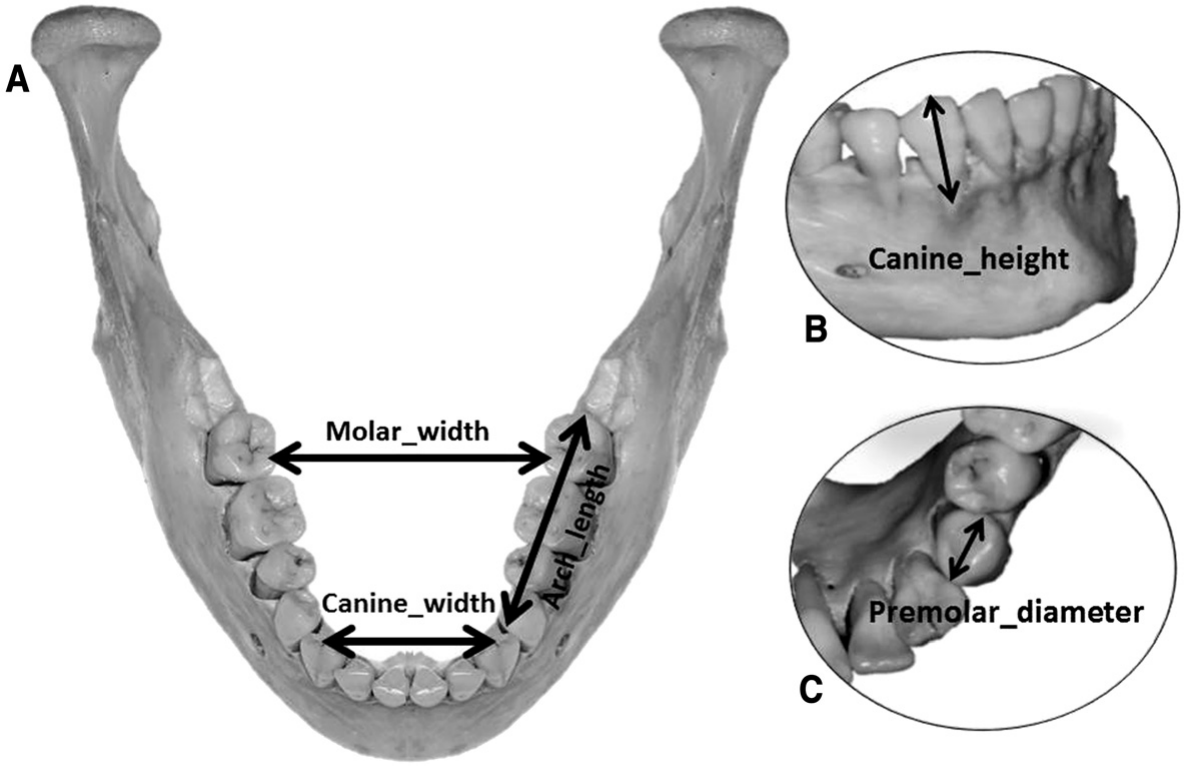
Five mandibular measurements made. **a** Occlusal view showing *intermolar width*, *intercanine width*, and *arch length*. **b** Partial view of the right *Canine_height*. **c** Partial view of the left *Premolar_diameter*

The STL files were imported into 3D Tool™ (version 10, Weinheim, Germany) software, the mandibles were reconstructed three-dimensionally, and five different measurements were made (Fig. 1). Intermolar width (Molar_width) was measured between the mesio-lingual cusp tips of the second molars. Intercanine width (Canine_width) was measured between the cusp tips of the right and left canines. Posterior arch length (Arch_length) was measured from the mesial of the first premolar to the distal of the second molar on the left side. Premolar crown diameter (Premolar_diameter) was measured between the tooth’s mesial and distal contact points on the left side. Canine height (Canine_height) was measured as the shortest distance from the buccal bone alveolar crest to the cusp tip of the right canine. Each measurement was made twice by one examiner (GDW), so that reliability of each of the three scanning protocols could be estimated. The same five measurements were also made on the actual mandibles using calipers accurate to 0.01 mm.

### Statistical analysis

The skewness and kurtosis statistics indicated normal distributions. Intraobserver systematic errors between the replicate scans were described as mean differences and compared statistically with paired *t* tests. Intraobserver random error was estimated using intraclass correlation coefficients (ICCs) and method errors (√(Σd^2^/2n) [21]. In order to evaluate systematic differences between scanners, the replicate measurements of each scanner were averaged. The systematic differences between scanners were described with means. Differences between scanners and differences between scanners and the actual mandibles were assessed using paired *t* tests. All statistical procedures were performed using IBM SPSS™ software (version 22.0, SPSS, Armonk, NY) using a significance level of 0.05.

## Results

Intraobserver systematic errors of the three scanners were similar (Table 1). Of the 15 differences, only one was statistically significant (*p* = .043). The first replicate of intermolar width (Molar_width) taken from the Ortho Insight 3D scans was 0.161 mm larger than the second replicate.

**Table 1:** Intraobserver systematic errors (mm) between the first and second replicates for each of the three scanning protocols, along with significances (Sig)

	Ortho Insight 3D		ITero		Lythos	
Measure	Difference (SD)	Sig	Difference (SD)	Sig	Difference (SD)	Sig
Molar_width	0.161 (0.279)	*0.043*	0.019 (0.221)	0.745	−0.002 (0.094)	0.938
Canine_width	0.045 (0.333)	0.606	0.017 (0.129)	0.628	0.002 (0.057)	0.872
Arch_length	0.045 (0.358)	0.632	0.008 (0.129)	0.820	−0.067 (0.153)	0.113
Premolar_diameter	−0.110 (0.204)	0.056	−0.004 (0.043)	0.756	0.003 (0.060)	0.852
Canine_height	−0.070 (0.211)	0.220	−0.049 (0.182)	0.316	0.005 (0.059)	0.760

Italic indicates statistically significant differences between replicates (*p* < .05)

Method errors ranged from 0.030 to 0.247 mm (Table 2). They were consistently larger with Ortho Insight 3D than with the two intraoral scanning methods. Method errors for the intermolar and intercanine width measurements and canine crown height were slightly smaller with Lythos than ITero. In contrast, methods errors for arch length and mesiodistal premolar width were larger with Lythos than ITero. Interclass correlations (ICC), ranging from .926 to .999, were consistently high and showed the same pattern of differences between scanning methods as did the method errors.

**Table 2:** Intraobserver random errors between replicates estimated with method errors (ME) and interclass correlations (ICC)

	Ortho Insight 3D		ITero		Lythos	
Variable	ME (mm)	ICC	ME (mm)	ICC	ME (mm)	ICC
Molar_width	0.222	0.994	0.152	0.996	0.064	0.999
Canine_width	0.230	0.982	0.089	0.997	0.039	0.999
Arch_length	0.247	0.989	0.088	0.999	0.114	0.998
Premolar_diameter	0.160	0.926	0.030	0.996	0.041	0.993
Canine_height	0.153	0.995	0.129	0.996	0.040	0.999

**Fig. 2:**
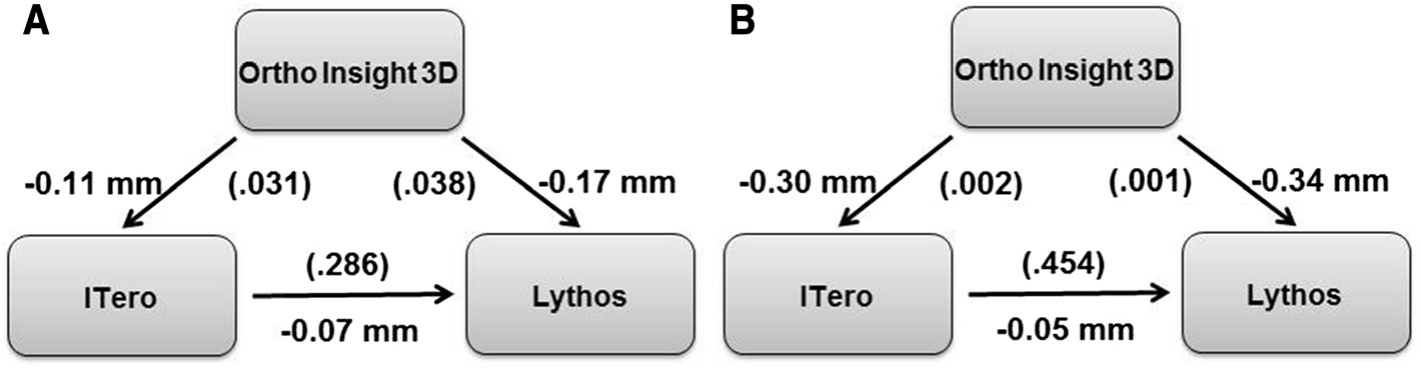
**a** Arch_length and **b** Canine_height comparisons among the scanners, with *arrows* pointing to the larger measure and probabilities in *parentheses*

Comparisons among the three scanners showed four statistically significant systematic differences (Fig. 2). Arch_length and Canine_height were significantly smaller when measured on the Ortho Insight 3D than on the other two scanners.

When compared to measurement made directly on the dried human mandibles, only Ortho Insight 3D showed statistically significant differences (Table 3). Arch_length (0.159 mm) and Canine_height (0.363 mm) were significantly smaller when measured on the Ortho Insight 3D reconstructions than when measured on the mandible.

**Table 3:** Systematic differences (mm) between the measures made directly on the dry mandibles and corresponding measures made on the 3D digital reconstructions for each of the three scanning protocols, with positive values indicating digital underestimation

	Ortho Insight 3D		ITero		Lythos	
Variable	Difference (SD)	Sig	Difference (SD)	Sig	Difference (SD)	Sig
Molar_width	−0.067 (0.314)	0.420	−0.012 (0.090)	0.613	0.016 (0.049)	0.230
Canine_width	−0.006 (0.164)	0.887	−0.035 (0.085)	0.132	−0.002 (0.045)	0.864
Arch_length	0.159 (0.275)	*0.042*	0.054 (0.219)	0.357	−0.012 (0.092)	0.613
Premolar_diameter	−0.064 (0.156)	0.136	−0.032 (0.077)	0.134	−0.005 (0.024)	0.424
Canine_height	0.363 (0.331)	*0.001*	0.066 (0.290)	0.391	0.018 (0.058)	0.252

Italic indicates statistically significant differences between replicates (*p* < .05)

## Discussion

Each of the three scanners produced accurate representations, with no consistent pattern of systematic errors. One of the Ortho Insight 3D measures showed statistically significant differences (0.161 mm) between replicate measurements. Importantly, the systematic errors in the present study were close to errors previously reported (ranging from −0.10 to 0.25 mm) for similar measurements [18, 19]. Measurement differences less than 0.20 mm have been suggested to be clinically acceptable [22]. If the individual has been adequately calibrated and maintains the same landmark definitions, systematic intraobserver differences should not be expected to occur.

All three scanners were also highly reliable, with ICCs ranging from 0.926 to 0.999. A previous study evaluating Ortho Insight 3D showed similar ICCs (95–96 %), which were higher than the ICCs associated with digital models generated with emodel system (GeoDigm, Chanhassen, Minn) and cone-beam computerized tomography [23]. Based on virtual models generated from CT scans, replicates showed ICCs ranging between 0.913 and 0.999 [24]. Considering that reliability coefficients above 0.75 have been considered to be excellent [25], the substantially higher ICCs obtained in the present study indicate excellent reproducibility.

**Fig. 3:**
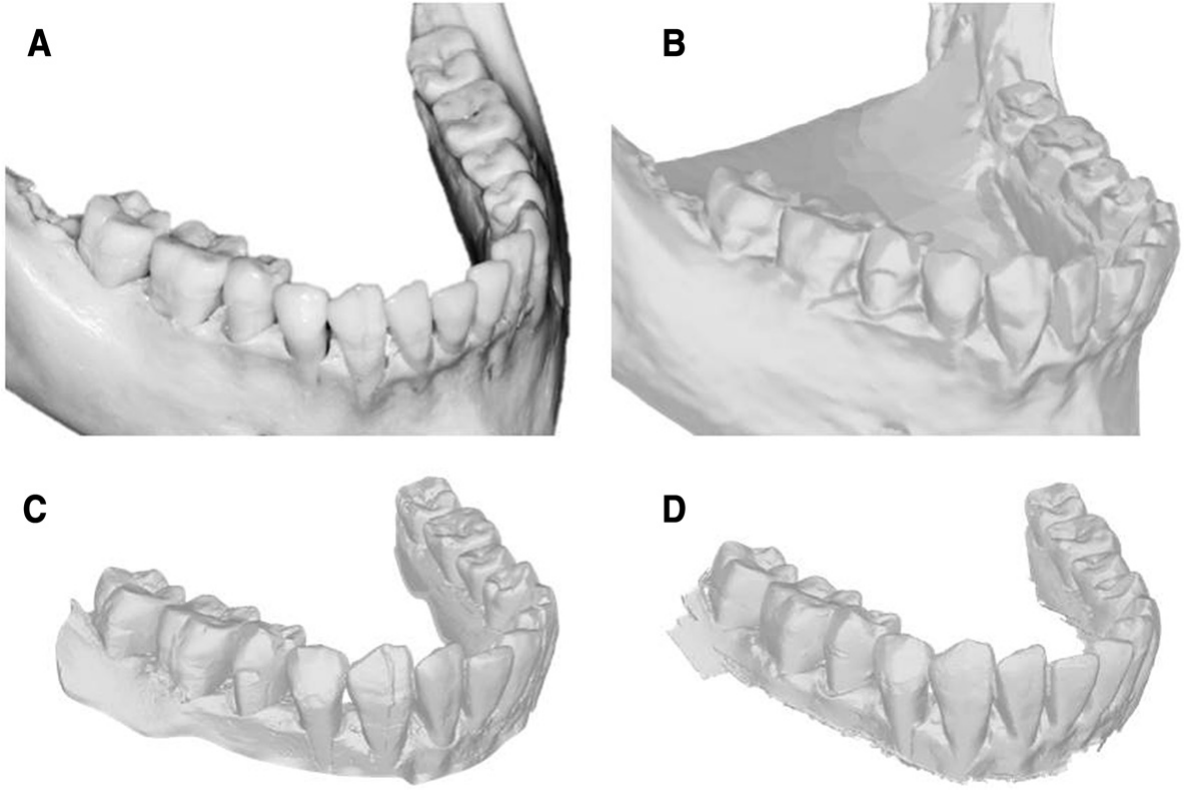
**a** Dry mandible. **b** Digital model generated by Ortho Insight 3D. **c** Digital model generated by ITero. **d** Digital model generated by Lythos

While they were all reliable, Ortho Insight 3D produced larger random errors than the two intraoral scanners. Arch_length measured with Ortho Insight 3D showed the greatest method error, with differences between replicate measurements differing by ±0.48 mm 95 % of the time. In contrast, the same measurement made from the ITero scanner varied by ±0.17 mm 95 % of the time. Ortho Insight 3D produced larger random measurement errors than the intraoral scanners because the image resolution was not as sharp, making landmark identification more difficult (Fig. 3). The method errors for premolar diameter in the present study were similar to or smaller than the length estimates obtained from OrthoCAD™ (Cadent Inc, Fairview, NJ) [26]. Method errors for intercanine width were also smaller than previously reported for measurements taken from O3DM™ (OrtoLab, Czestochowa, Poland) [27] and 3Shape (D-250; 3Shape, Copenhagen, Denmark) [13] scans.

Measurements taken from the mandibles scanned with ITero and Lythos compared closely to the same measurements taken directly from the dry mandibles. Most of measurements were comparable, with average differences ranging between .002 and .066 mm. While the Lythos scans have not been previously evaluated, ITero scans have been previously shown to be highly accurate [17]. The results indicate that both scanners produce valid presentations of the mandible.

Two of the measurements taken from the Ortho Insight 3D scan reconstructions were slightly smaller than the corresponding measures taken on the dry mandibles. Comparing plaster models and emodels, Mullen and coworkers [28] also found significant differences, with arch lengths measured on plaster models being approximately 1.5 mm larger than arch lengths measure on emodels. Using an extraoral scanner (Optimet 3D scanner), Redlich and coworkers [29] also reported statistically significant differences in mandibular arch length measurements obtained from plaster and digital models. Another study comparing plaster models and emodels found significant differences in anterior mandibular arch perimeter (plaster model measurements were 0.40 mm larger), but no differences when perimeter included all of the teeth between the first mandibular molars [10]. Schirmer and Wiltsire [22] attributed the differences between digital and actual models to the difficulty of measuring a 3D object in two dimensions, i.e., on a computer monitor.

The results of this study were probably limited by the use of dry mandibles. Because measurements on dry mandibles can be more easily standardized, they might be expected to be more reliable than the same measurements taken in vivo. This could explain why intraoral scanning with the ITero is less precise than extraoral scanning with ITero [20]. It would also have been possible to reduce random error by marking the landmarks on the mandibles prior to scanning them, which was not done because we wanted more realistic error estimates.

## Conclusions

Within the limits of this study (in vitro tests conducted on dry mandibles), the results showed that

1. Measurements made from digital models produced by Ortho Insight 3D, ITero, and Lythos were highly reliable;
2. While there are no systematic differences between measurements taken direction on dry mandibles and corresponding measures taken from ITero and/or Lythos scans, Ortho Insight 3D scans (<.36 mm) slightly underestimated Arch_length and Canine_height.
